# Inhibition of PKC disrupts addiction-related memory

**DOI:** 10.3389/fnbeh.2014.00070

**Published:** 2014-03-07

**Authors:** Kristin K. Howell, Bradley R. Monk, Stephanie A. Carmack, Oliver D. Mrowczynski, Robert E. Clark, Stephan G. Anagnostaras

**Affiliations:** ^1^Molecular Cognition Laboratory, Department of Psychology, University of CaliforniaSan Diego, La Jolla, CA, USA; ^2^Division of Biological Sciences, University of CaliforniaSan Diego, La Jolla, CA, USA; ^3^Veterans Affairs Medical CenterSan Diego, CA, USA; ^4^Department of Psychiatry, University of CaliforniaSan Diego, La Jolla, CA, USA; ^5^Program in Neurosciences, University of CaliforniaSan Diego, La Jolla, CA, USA

**Keywords:** PKC, cocaine, sensitization, memory, nonassociative, ZIP

## Abstract

The atypical PKC isoforms, PKMζ and PKCλ have been proposed as integral substrates of long-term memory (LTM). Inhibition of these isoforms has recently been demonstrated to be sufficient for impairing the expression and maintenance of long-term potentiation. Additionally, the pseudosubstrate inhibitor, zeta inhibitory peptide (ZIP), which effectively blocks PKMζ and PKCλ, has previously been shown to disrupt associative memory; very little is known about its effects on pathological nonassociative forms of memory related to addiction. The neural and molecular substrates of memory and addiction have recently been argued to overlap. Here, we used ZIP to disrupt PKMζ and PKCλ activity to examine their role in cocaine sensitization, a nonassociative, addiction-related memory argued to underlie the transition from casual to pathological drug use. We examined the effects of both continuous and acute administration of ZIP. Even a single application of ZIP blocked the development of sensitization; sustained inhibition using osmotic pumps produced an almost complete blockade of sensitization. Further, a single application of ZIP was shown to reduce membrane-bound AMPAR expression. These results demonstrate a novel, critical role for the atypical PKC isoforms in nonassociative memory and cocaine addiction.

## Introduction

Addiction involves long-lasting behavioral and neural changes thought to render the addict chronically susceptible to relapse (Robinson and Kolb, 1997; Robbins and Everitt, 1999; Nestler, 2001; Hyman et al., 2006; Koob and Volkow, 2010; Russo et al., 2010; Lüscher and Malenka, 2011). Recently, it has been proposed that the mechanisms of learning and memory, and addiction overlap and that memory or memory-like neuronal remodeling subserve addiction (Kelley, 2004; Kauer and Malenka, 2007; Robinson and Berridge, 2008; Russo et al., 2010; Torregrossa et al., 2011; Carmack et al., 2013).

In both processes, these changes involve the activation of multiple protein kinases including CaMKIIα, PKA, and PKC (Mayford, 2007; Lee and Messing, 2008; Kandel, 2012; Lisman et al., 2012). Recently, there has been growing evidence specifically implicating atypical isoforms of PKC (aPKCs) in LTP and memory (Pastalkova et al., 2006; Shema et al., 2007; Sacktor, 2008; Serrano et al., 2008; Ren et al., 2013). One isoform that has received much attention is protein kinase Mζ (PKMζ). PKMζ is persistently active and lacks the regulatory domain present on most protein kinases, giving rise to the idea that PKMζ may be essential for long-term memory (LTM) and late long-term potentiation (L-LTP). An abundance of studies implicating PKMζ in LTM and L-LTP currently exists (Pastalkova et al., 2006; Shema et al., 2007; Serrano et al., 2008; Kwapis et al., 2009, 2012; Parsons and Davis, 2011). However, recent studies using PKCζ /PKMζ knockout mice have questioned the idea that PKMζ is necessary and sufficient for L-LTP and LTM and proposed that a second atypical PKC isoform, PKCλ, is involved, especially in early stages (Frankland and Josselyn, 2013; Lee et al., 2013; Matt and Hell, 2013; Ren et al., 2013; Volk et al., 2013).

Evidence implicating PKMζ in LTM and L-LTP comes from studies using a single application of zeta-inhibitory peptide (ZIP) or the more general PKC inhibitor chelerythrine (Herbert et al., 1990; Yao et al., 2013). A few studies have used the dominant negative form of PKMζ to inhibit PKMζ and subsequently disrupt LTP (Ling et al., 2002) and established memory (Shema et al., 2007). Once believed to be selective to PKMζ, ZIP was recently shown to also inhibit PKCλ (Ren et al., 2013). ZIP is derived from the autoinhibitory pseudosubstrate segment of PKCζ, which is the same as that of PKCλ (Standaert et al., 2001; Bosch et al., 2004). Thus, the effects of ZIP may result from PKMζ and/or PKCλ inhibition. For the current studies, it is only important that plasticity and associative memory are disrupted by ZIP.

While many prior studies have demonstrated the efficacy of ZIP in impairing both associative memory and L-LTP (Pastalkova et al., 2006; Shema et al., 2007; Serrano et al., 2008; Kwapis et al., 2009; Parsons and Davis, 2011; Barry et al., 2012), the role of aPKCs in addiction-related memory is unclear. We examined if ZIP disrupts the nonassociative, addiction-related memory, psychomotor sensitization to cocaine. Sensitization is an enhanced sensitivity to a drug characterized by increased psychomotor activation (locomotor sensitization), increased dopamine release (neural sensitization), and hypersensitivity to the drug's rewarding value (incentive sensitization) and is used to model the transition from casual to compulsive drug use (Robinson and Berridge, 1993; Anagnostaras and Robinson, 1996; Anagnostaras et al., 2002; Steketee and Kalivas, 2011; Shuman et al., 2012). Thus, this memory is thought to reflect pathological and compulsive behavior rather than ordinary associative learning.

Cocaine has been shown to change properties associated with excitatory synaptic transmission. Both in slice and *in vivo*, alterations in AMPAR/NMDAR ratios and increases in AMPAR rectification have been demonstrated following cocaine treatment (Kauer and Malenka, 2007; Kessels and Malinow, 2009). PKMζ and PKCλ may exert their effects through AMPAR trafficking (Ling et al., 2006; Yao et al., 2008; Migues et al., 2010; Sacktor, 2011; Ren et al., 2013). Perfusion of PKMζ into cells doubled the AMPA mediated EPSC and inhibition of PKMζ decreased post-synaptic GluR2 (Sacktor, 2008; Migues et al., 2010). Similarly, PKCλ inhibition blocked the enhancement of GluA1/GluA2 typically induced by LTP suggesting the elevation of post-synaptic AMPARs is dependent on PKCλ activity (Ren et al., 2013). Thus, the aPKCs, PKMζ and PKCλ may also mediate modifications in AMPARs during addiction-related memory and plasticity.

We examined the effects of disruption of aPKCs at multiple time points during the induction of sensitization using continuous or acute intracerebroventricular (ICV) application of ZIP or chelerythrine. Finally, we examined whether acute disruption of PKMζ reduced membrane-bound AMPAR density.

## Materials and methods

### Animals

Ninety-six hybrid C57BL/6Jx129T2SvEms/J (129B6, Jackson Labs) adult mice were used. Mice were group housed in a vivarium on a 14:10 light:dark schedule. Testing was performed during the light phase. All procedures were approved by the UCSD IACUC and compliant with the NRC Guide.

### Drugs

The myristolated PKC Zeta pseudosubstrate inhibitory peptide (AnaSpec) was dissolved in phosphate-buffered saline (PBS, Ricca) to a dose of 10 nmol. In Experiment 1, 10 nmol of ZIP was administered across 3 days at a rate of 0.25 μ L/hr whereas in Experiments 3 and 4, the 10 nmol dose of ZIP was given in a single 1 μ L infusion. Chelerythrine Cl (Enzo) was dissolved in PBS to a concentration of 10 nmol/μ L. Buprenorphine HCl (0.05 mg/kg, s.c.) was given for post-operative pain (Reckitt-Benckiser). Cocaine HCl (Sigma) was dissolved in physiological saline (salt weight, 15 mg/kg, 10 ml/kg, i.p.).

### Surgery

For all experiments mice were anesthetized with isoflurane dispensed from a precision vaporizer and mounted in a stereotaxic apparatus (myNeuroLab.com). A single hole was drilled in the skull for infusion into the third ventricle (AP: −0.5 mm; ML: 0 mm, DV: −3 mm, Franklin and Paxinos, 2007). Following surgery, all animals were given an injection of buprenorphine. For experiments examining continuous inhibition of aPKCs (Experiments 1, 2), osmotic pumps (Alzet-Durect model 1002) and PE60 tubing were implanted subcutaneously and connected to an infusion headstage attached to the skull (Alzet, Brain infusion kit 3).

#### Experiments 1 and 2

**Figure 1:**
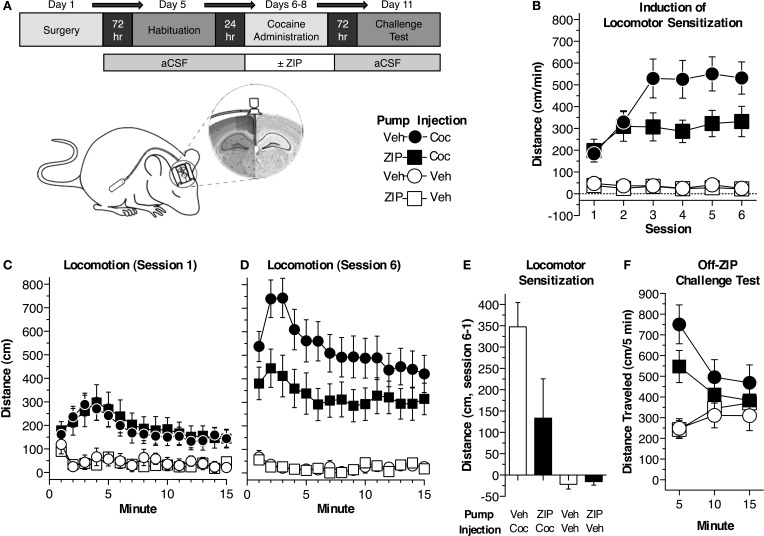
**Continuous ZIP administration blocks cocaine-induced locomotor sensitization. (A)** Depiction of the procedure used in experiment 1 (*n* = 10 Veh/Coc, *n* = 11 ZIP/Coc, *n* = 7 Veh/Veh, *n* = 8 ZIP/Veh). Mice were implanted with osmotic pumps, filled such that ZIP would be delivered ICV just prior to the first cocaine administration session and terminate 24 h following the sixth session (10 nmol, 0.25 μL/h). Animals underwent six cocaine administration sessions (2 sessions per day) during which time the development and expression of sensitization was assessed. **(B)** ZIP impaired the development of sensitization. The data represent the average distance traveled for each session (±s.e.m.). The distance traveled by mice receiving ZIP/Coc was reduced compared to mice treated with Veh/Coc across the six cocaine administration sessions [15 mg/kg, i.p.; MANOVA, *F*_(3, 32)_ = 18.3, *p* < 0.001; Fisher's PLSD *p* < 0.05]. ZIP alone did not produce any locomotor attenuating effects (ZIP/Veh, Veh/Veh, *p* = 0.93). **(C)** ZIP did not alter the acute response to cocaine (session 1). Distance traveled at each minute of the session (±s.e.m.) is shown. Animals receiving cocaine (ZIP/Coc, Veh/Coc) traveled a greater distance compared to mice receiving vehicle (ZIP/Veh, Veh/Veh, *p* < 0.05), but there was no difference between the cocaine-treated groups (*p* = 0.78). **(D)** ZIP/Coc mice showed reduced locomotor sensitization compared to Veh/Coc mice during session 6 [ANOVA, *F*_(3, 32)_ = 16.7, *p* < 0.001; ZIP/Coc, Veh/Coc Fisher's PLSD *p* < 0.05]. Distance traveled at each minute of the session (±s.e.m.) is shown. ZIP alone did not produce any effects on locomotor activity. **(E)** Sensitization, measured as the difference between the sensitized (session 6) and acute (session 1) responses, was blocked in ZIP/Coc mice. The average difference in distance traveled (±s.e.m.) is shown for each group. Sensitization in ZIP/Coc mice did not differ from mice treated with ZIP/Veh or Veh/Veh (*p* > 0.1). **(F)** Sensitization was also assessed while all animals were off-ZIP. ZIP/Coc and Veh/Coc groups showed greater activity than mice that previously received vehicle [ZIP/Veh, Veh/Veh; MANOVA, *F*_(3, 32)_ = 3.2, *p* < 0.05]. Animals in the ZIP/Coc group showed reduced sensitization compared to animals that had previously received Veh/Coc during the first 5 min of the test [ANOVA, *F*_(3, 32)_ = 10.373, *p* < 0.001; Fisher's PLSD, ZIP/Coc vs. Veh/Coc, *p* < 0.05]. There were no differences in the acute response to cocaine between animals that had not previously received cocaine (Fisher's PLSD, Veh/Veh, ZIP/Veh, *p* = 0.96).

In Experiment 1, 16 h prior to surgery, pumps were filled with aCSF (100 μ L; ion concentrations in mM: Na 150, K 3.0, Ca 1.4, Mg 0.8, P 1.0, Cl 155; Harvard) and connected to tubing containing ZIP and/or aCSF. A “leader” and “trailer” of aCSF was placed before and after the ZIP in the tubing (separated with mineral oil) timed such that ZIP administration began 8.5 h prior to the beginning of cocaine administration, and ended 23 h after the sixth cocaine administration session (Figure 1A). In Experiment 2, pumps and tubing were filled with chelerythrine or aCSF. Animals recovered for 3 days.

#### Experiments 3 and 4

In Experiment 3, mice were given one microinfusion of ZIP prior to the induction of sensitization. Mice were implanted with 20-ga guide cannulae (PlasticsOne) 1 mm above the target. A dummy was placed inside the guide to prevent clogging. Animals recovered for 3 days. Prior to ZIP infusion, mice were briefly anaesthetized with isoflurane. Dummies were removed and a 24-ga injection cannula that extended 1 mm below the guide was attached. ZIP was infused at a rate of 1 μ L/min using a syringe pump (Kd Scientific) and injection cannulas were left in place for 3 min following the infusion. Animals recovered for 2 h. In Experiment 4, after the induction of sensitization, mice were given an infusion of ZIP or aCSF. A 29-ga stainless steel needle connected to a syringe and pump infused ZIP at a rate of 1 μ L/min. The needle remained in place for 3 additional min. Animals recovered for 24 h.

### Behavioral assessment

Mice were tested in individual chambers housed in a windowless room as described previously (Shuman et al., 2012; Carmack et al., 2013). The apparatus consisted of a two-sided, 44 × 44 × 31 cm chamber, bisected by an opaque wall with a removable insert (Med-Associates). Sides assigned for drug and saline pairings were counterbalanced. Activity monitor software (Med-Associates) tracked the distance traveled. Two 100-watt bulbs lit the room and white noise (65 dBA) played continuously. All animals were handled for 5 days prior to the experiments. Prior to behavioral assessment, animals were habituated to the chambers during two, 1 h sessions (30 min each side).

#### Experiment 1

Mice were divided into four groups: Veh/Coc, ZIP/Coc, Veh/Veh, ZIP/Veh (Figure 1A). Sensitization was induced during six sessions of cocaine administration (2 sessions/day for 3 days). Animals received an injection of saline (10 ml/kg) and were immediately placed in the saline-paired side of the chamber. Animals were restricted to this side for 15 min. Mice were then removed, given an injection of cocaine (Veh/Veh and ZIP/Veh mice received a second injection of saline) and restricted to the drug-paired side of the chamber. After 15 min, mice were removed from the chambers and returned to their home cages. ZIP was infused continuously throughout the six cocaine administration sessions. Forty eight hours following the 6th cocaine administration session, animals underwent a conditioned place preference test. All animals were off-ZIP and off-drug. The insert bisecting the two sides of the chamber was removed and animals were allowed to freely explore both sides of the chamber for 15 min. Place preference was measured as the difference in percent time spent on the drug-paired side and saline-paired side. A final, off-ZIP sensitization challenge test was conducted 24 h later. All animals were given an injection of cocaine (15 mg/kg) and were immediately placed on the drug-paired side of the chamber. Animals were restricted to the drug-paired side and remained in the chambers for 15 min. Sensitization was measured as the increase in locomotor activity following repeated drug-context pairings.

#### Experiment 2

Chelerythrine was delivered continuously throughout the entire experiment. Procedures were the same as those described above; however, in this experiment, animals were given five cocaine administration sessions across 5 days, followed by a place preference test 24 h later and the sensitization challenge test another 24 h later (session 6).

#### Experiment 3

Mice were infused with ZIP (described above) 2 h prior to the first cocaine administration session (session 1). 24 h later mice were given an off-drug place preference test. Another 24 h later, mice were given a sensitization challenge test (session 2).

#### Experiment 4

Animals were given four cocaine administration sessions across 4 days. 24 h after the final session, animals underwent surgery and were infused with ZIP or vehicle (described above). Following recovery, animals were given an off-drug place preference test followed 24 h later by a sensitization challenge test (session 5).

### Histology

In Experiments 1 and 2 mice were anaesthetized and perfused with 1 × PBS and 4% paraformaldehyde. Tissue was sliced into 1 mm coronal sections using an acrylic matrix (Braintree). For Experiments 3 and 4, animals were anesthetized and then decapitated for fresh tissue collection. Brains were extracted, frozen in 2-methylbutene and stored at −80°C. At −20°C brains were cut in 20 μm coronal sections at 200 μm intervals using a cryostat (Microm HM550, Fisher). Cannula placement was verified by visual inspection. Brain morphology remained grossly intact following the application of ZIP and chelerythrine. No animals were excluded.

### Radioligand incubation and liquid scintillation

Optimal binding procedures for the [^3^H]AMPA radioligand are adapted from previous literature (Olsen et al., 1987; Jang et al., 2000; Monk et al., 2012). Sections were pre-incubated with 50 mM Tris-HCl buffer for 20 min, then incubated for 30 min with 15 nM [^3^H]AMPA (Sigma) in the same buffer at 25°C. After incubation, the sections were rinsed in the Tris-HCl buffer, then washed in distilled water. Sections from each slide were transferred to vials containing a liquid scintillation cocktail (EcoLume Liquid Scintillation Fluid, MPBiomedicals) to assess global AMPA receptor expression density using automated liquid scintillation (Tricarb 2900TR, PerkinElmer).

### Data analysis

Data were entered into a multivariate analysis of variance (MANOVA; PASW18). The level of significance was set at *p* ≤ 0.05. Following a significant omnibus comparison, or group x time interaction, *post-hoc* comparisons were made using univariate ANOVAs or Fisher's protected least significant difference (PLSD). In order to simplify data presentation, univariate ANOVAs are reported for group differences, followed by Fisher's PLSDs for interesting comparisons.

## Results

### Experiment 1: effects of continuous ZIP administration on locomotor sensitization

We first examined the effects of continuous aPKC inhibition on psychomotor sensitization to cocaine. Mice were implanted with osmotic pumps that delivered continuous, ICV ZIP or aCSF (10 nmol, 0.25 μL/h) throughout six cocaine (15 mg/kg, i.p.) or saline administration sessions (Figures 1A,B). ZIP administration was timed such that it began prior to the first cocaine administration session and ended after the sixth session. Mice were divided into four groups (*n* = 7–11 per group): (1) Veh/Coc mice received vehicle, ICV, in the pumps and i.p. cocaine injections, (2) ZIP/Coc mice were administered ZIP ICV through pumps and received i.p. cocaine injections, (3) Veh/Veh mice received vehicle both ICV and i.p., (4) ZIP/Veh mice received ICV ZIP through the pumps, but received i.p. injections of vehicle. We found an initial elevated locomotor response in groups receiving cocaine compared to those receiving vehicle, [Figure 1C; ANOVA, *F*_(3, 32)_= 4.6, *p* = 0.009], but no difference in the acute response to cocaine between ZIP/Coc mice and Veh/Coc mice (Fisher's PLSD, *p* = 0.78). Across the six sessions of cocaine administration, differences between ZIP/Coc and Veh/Coc mice emerged [Figures 1B,D,F; *F*_(3, 32)_ = 18.3, *p* < 0.001]. ZIP/Coc mice demonstrated a dramatic reduction in locomotor activity compared to Veh/Coc mice (*p* < 0.05). When paired with saline, ZIP did not produce any locomotor attenuating effects (ZIP/Veh vs. Veh/Veh, *p* = 0.93). We then measured sensitization as the difference between the acute (session 1) and sensitized (session 6) response (Shuman et al., 2012; Carmack et al., 2013). There were significant group differences [*F*_(3, 32)_ = 6.9, *p* = 0.001; Figure 1E]; Veh/Coc mice exhibited robust sensitization, showing a greater response than all other groups (*p* < 0.02). Sensitization was blocked in ZIP/Coc mice, as they did not differ from control groups (*p* > 0.1; Figure 1E). We conducted a final sensitization challenge test, during which all animals were off-ZIP and all groups received cocaine (15 mg/kg, i.p.; Figure 1F). Groups that had previously received cocaine (ZIP/Coc, Veh/Coc) showed greater activity compared to groups that had previously received vehicle [ZIP/Veh, Veh/Veh; *F*_(3, 32)_ = 3.2, *p* < 0.05; Figure 1F], but ZIP/Coc mice showed attenuated sensitization relative to Veh/Coc mice [first 5 min, main effect, *F*_(3, 32)_ = 10.4, *p* < 0.001, ZIP/Coc vs. Veh/Coc, *p* < 0.05; Figure 1F].

### Experiment 2: effects of continuous chelerythrine administration on locomotor sensitization

**Figure 2:**
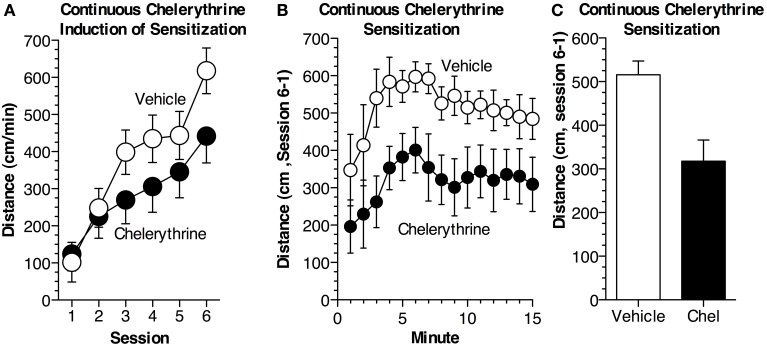
**Continuous chelerythrine reduces locomotor sensitization. (A)** Mice were implanted with osmotic pumps containing chelerythrine or vehicle. Chelerythrine (*n* = 9 Chel, *n* = 8 Veh) was delivered continuously, ICV (10 nmol/μL, 0.25 μL/h), across six sessions of cocaine administration (15 mg/kg, i.p.). Average distance traveled (±s.e.m.) during each session is depicted. Chelerythrine did not alter the acute response to cocaine [ANOVA, *F*_(1, 15)_ = 0.097, *p* = 0.76] but did reduce the development of sensitization across the six sessions. **(B)** Sensitization, measured as the difference in distance traveled between the acute (session 1) and sensitized (session 6) response was significantly impaired in mice receiving chelerythrine across 15 min [MANOVA, *F*_(1, 15)_ = 11.1, *p* < 0.01]. The difference in distance traveled (± s.e.m.) is shown for each minute. **(C)** Average sensitization (±s.e.m.) measured as the difference in distance traveled is shown for each group.

As we used a novel, chronic procedure to inhibit aPKCs, in Experiment 2, we investigated whether continuous chelerythrine administration would affect sensitization similarly to ZIP. Chelerythrine more generally blocks PKCs by competitively inhibiting the catalytic domain and effectively inhibits PKM isoforms (Herbert et al., 1990; Serrano et al., 2008; Yao et al., 2013). This experiment was conducted to validate the effectiveness of using osmotic minipumps and continuous delivery to inhibit aPKCs. Mice were implanted with osmotic pumps which delivered chelerythrine or vehicle throughout the experiment at a dose established by others (10 nmol/μL, 0.25 μL/h, Serrano et al., 2008; Yao et al., 2013). Mice were divided into two groups (*n* = 8–9 per group): (1) received chelerythrine (10 nmol/μL) ICV as well as i.p. cocaine injections and (2) vehicle mice received aCSF ICV and i.p. injections of cocaine. All animals underwent six cocaine administration sessions (15 mg/kg, i.p.; Figure 2A). As with ZIP, chelerythrine did not affect the acute response to cocaine during session 1 [*F*_(1, 15)_ = 0.1, *p* = 0.76]. After the final cocaine administration session, sensitization was assessed as the difference between the acute (session 1) and sensitized response (session 6). Sensitization was dramatically attenuated in mice previously treated with chelerythrine relative to mice that had received vehicle [Figures 2B,C; *F*_(1, 15)_ = 11.1, *p* < 0.01].

### Experiment 3: effects of acute, pre-induction ZIP on locomotor sensitization

**Figure 3:**
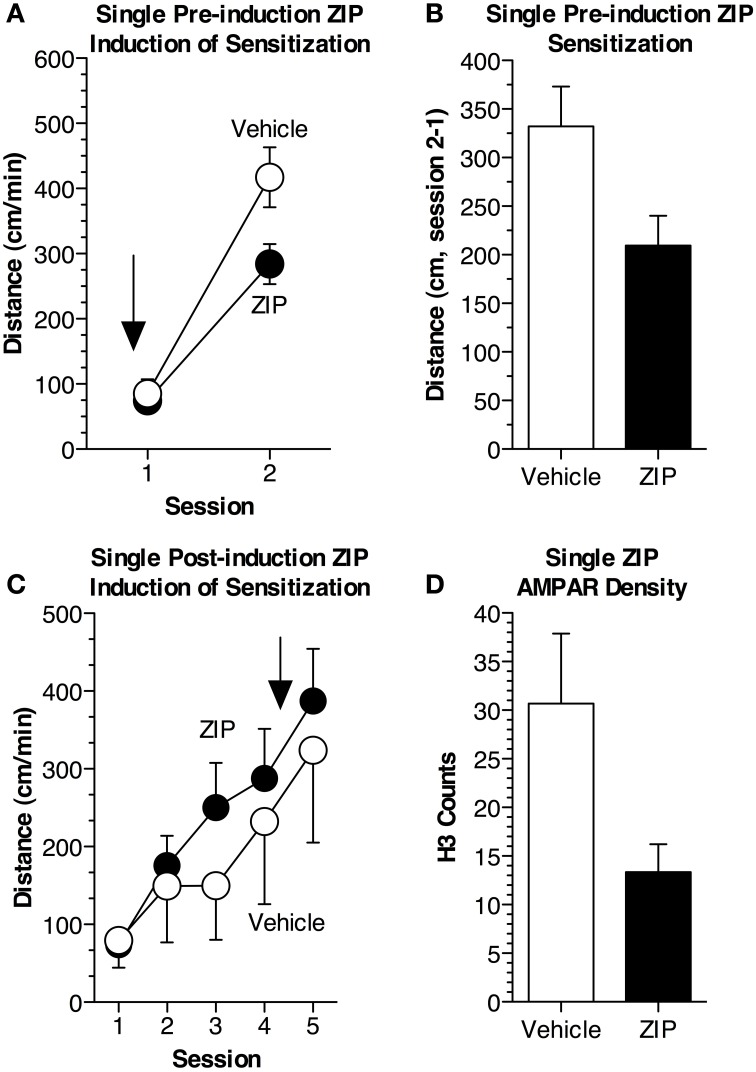
**Single pre- but not post-induction application of ZIP reduces locomotor sensitization. (A)** Mice received a single pre-induction infusion of ZIP (10 nmol/μL, 1 μL). Sensitization was induced during two sessions of cocaine administration (15 mg/kg, i.p.). ZIP was administered 2 h prior to the first cocaine administration session (*n* = 13 ZIP, *n* = 13 aCSF), indicated by the arrow. The average distance traveled (±s.e.m.) for each session is shown. While on-ZIP, the acute response to cocaine was not altered [ANOVA, *F*_(1, 24)_ = 0.215, *p* = 0.65]. Cocaine was given for a second time, 48 h later, during session 2. When measured off-ZIP, distance traveled was significantly reduced in animals that had previously received ZIP [ANOVA, *F*_(1, 24)_ = 5.8, *p* < 0.05]. **(B)** Sensitization was significantly reduced in animals given ZIP prior to cocaine administration [ANOVA, *F*_(1, 24)_ = 5.7, *p* < 0.05]. Sensitization is represented as the average difference in distance traveled (±s.e.m.) between the two sessions. **(C)** Mice received a single post-induction infusion of ZIP (10 nmol/μL, 1 μL). Sensitization was induced across 4 sessions of cocaine administration (15 mg/kg, i.p.), after which mice were given a single infusion of ZIP, represented by the arrow. Post-induction ZIP did not produce impairment when sensitization was assessed 72 h later off-ZIP (ZIP *n* = 9, Veh *n* = 8). **(D)** A single infusion of ZIP reduces AMPAR density following sensitization. H3 counts (±s.e.m.) for each group are depicted.

As most previous studies have given a single infusion of ZIP to assess the effects on memory, we examined if a single infusion could disrupt sensitization (Pastalkova et al., 2006; Shema et al., 2007; Serrano et al., 2008; Kwapis et al., 2009; Parsons and Davis, 2011). In this experiment, we used two groups of mice (*n* = 13 per group): (1) received a single pre-induction application of ZIP (10 nmol/μL, 1 μL) 2 h prior to the first cocaine administration session, while (2) received a 1 μL infusion of aCSF prior to cocaine administration (Figure 3A). During this initial session (15 mg/kg, i.p.), ZIP did not affect the response to cocaine [Figure 3A; *F*_(1, 24)_ = 0.22, *p* = 0.65]. In contrast, when challenged with cocaine while off-ZIP, 48 h later, mice that had previously received ZIP showed substantial impairment in sensitization [Figure 3A; *F*_(1, 24)_ = 5.8, *p* < 0.05]. Further, ZIP also impaired sensitization when assessed as the difference in activity between the two sessions [Figure 3B; *F*_(1, 24)_ = 5.7, *p* < 0.05].

### Experiment 4: effects of acute, post-induction ZIP on locomotor sensitization

We then examined whether a single, post-induction application of ZIP could disrupt locomotor sensitization. Two groups of mice were used (*n* = 8–9 per group); both groups received i.p. injections of cocaine (15 mg/kg, i.p.), but one group received an ICV infusion of ZIP while the other received an ICV infusion of aCSF. Four cocaine administration sessions produced robust sensitization that did not differ across groups [Figure 3C; *F*_(1, 15)_ = 0.29, *p* = 0.59]. After the fourth session, mice were given a single microinfusion of ZIP (10 nmol/μL, 1 μL) or a comparable infusion of aCSF. Forty-eight hours later, we conducted an off-ZIP sensitization test. Post-induction ZIP failed to affect sensitization [Figure 3C; *F*_(1, 15)_ = 0.23, *p* = 0.63].

### Experiment 5: effects of ZIP on AMPAR density

Finally, we examined whether a single application of ZIP was sufficient to reduce AMPAR density in sensitized brain tissue. As it has been argued that both PKMζ and PKCλ exert their effects through AMPAR trafficking (Ling et al., 2006; Yao et al., 2008; Migues et al., 2010; Ren et al., 2013), we used a radioligand binding procedure to detect membrane-bound [^3^H]AMPA. We found that membrane-bound AMPARs were significantly reduced in tissue previously exposed to ZIP [Figure 3D; *F*_(1, 34)_ = 6.2, *p* < 0.02].

## Discussion

In the present study, we examined the effects of both continuous and acute inhibition of the aPKC isoforms, PKMζ and PKCλ, on the nonassociative, addiction-related memory, locomotor sensitization. There were two main findings. First, aPKCs are critically involved in the development of locomotor sensitization; ZIP was highly effective at disrupting sensitization if infused prior to cocaine administration. Second, infusion of ZIP after sensitization had been established failed to produce impairment, despite reducing the density of membrane-bound AMPARs. The current findings extend the existing evidence regarding which forms of memory are susceptible to disruption by ZIP. The novel method for ZIP administration reveals nonassociative memory may have different requirements for memory maintenance than traditional forms of memory, as pre-induction ZIP was required to produce impairment.

### ZIP administration disrupts the development of sensitization

Growing evidence supports the view that ZIP disrupts not only PKMζ, but also a second atypical PKC isoform, PKCλ (Lee et al., 2013; Ren et al., 2013; Volk et al., 2013). The majority of studies have used a single application to disrupt associative or spatial forms of memory such as conditioned taste aversion, Pavlovian fear conditioning, fear potentiated startle, and the Morris water maze (Pastalkova et al., 2006; Shema et al., 2007; Serrano et al., 2008; Kwapis et al., 2009; Parsons and Davis, 2011). ZIP is derived from the pseudosubstrate sequence of PKCζ, which is identical to that of PKCλ. At higher concentrations, ZIP inhibits both PKMζ and PKCλ (Standaert et al., 2001; Bosch et al., 2004; Ren et al., 2013). Here, we expand current findings to include a role for PKMζ and PKCλ in the nonassociative, addiction-related memory, locomotor sensitization. Administration of either ZIP or chelerythrine prior to induction impaired the development of sensitization. ZIP dramatically impaired sensitization regardless of whether it was given continuously or in a single infusion, but the effects were largest when given continuously (Figures 1B,E). Still, a single application of ZIP disrupted sensitization 48 h after administration (Figures 3A,B), a time point when ZIP would have been fully degraded (Kwapis et al., 2012). These results demonstrate ZIP persistently effects sensitization if administration occurs prior to acquisition.

There are a few previous reports demonstrating ZIP's ability to disrupt certain forms of addiction-related plasticity and memory including cocaine-induced spontaneous synaptic transmission, the cocaine-induced enhancement in AMPA/NMDA ratio and conditioned place preference (Li et al., 2011; Ho et al., 2012). However, these studies focused on associative forms of addiction related memory. The present study is the first to demonstrate its ability to disrupt nonassociative addiction related memory. Nonassociative aspects of addiction are important to consider as they model key pathological components of what drive addiction.

### ZIP does not impair the maintenance of sensitization

Studies that have examined the application of post-training ZIP have found that it often produces amnesia (Pastalkova et al., 2006; Shema et al., 2007; Serrano et al., 2008; Kwapis et al., 2009; Gámiz and Gallo, 2011). However, Parsons and Davis (2011) suggested the effects of ZIP were dependent on the timing between training and administration. While memory and addiction have been argued to share overlapping neural substrates (Robinson and Kolb, 1997; Kelley, 2004; Kauer and Malenka, 2007; Lee and Messing, 2008), findings from the current study suggest the role of PKMζ and PKCλ in sensitization differs somewhat from their role in associative memory. Once sensitization had been established, ZIP administration was unable to produce subsequent impairment (Figure 3C). Moreover, sustained inhibition was required to fully prevent sensitization.

One possible reason that may account for these differences is the region-specificity of the infusion. Most prior studies have infused ZIP into a particular region (e.g., amygdala, insular cortex, hippocampus), however we administered ZIP ICV. It is possible that the concentration of ZIP required to produce an effect after sensitization had been established was not achieved. Previous work has established that a certain concentration of ZIP is required to block PKMζ and PKCλ and impair plasticity (Serrano et al., 2005; Sacktor and Fenton, 2012; Ren et al., 2013). A similar explanation could potentially account for differences in the effects of ZIP on conditioned place preference found in this study compared to other studies that have shown the apparent erasure of CPP memory following the administration of PKMζ inhibitors (Supplementary Figure S1, He et al., 2011; Li et al., 2011; Shabashov et al., 2012; Lee et al., 2013). It is also possible that by infusing ZIP ICV the peptide did not reach regions critical for the behavior, such as the amygdala (Everitt et al., 1991; Hsu et al., 2002; He et al., 2011). While this explanation may explain the negative result in our place preference experiments, it likely does not account for our sensitization results because the concentration achieved in the current study was sufficient to disrupt sensitization prior to induction and produced a decrease in AMPAR density when given post-induction.

An alternate reason we did not find an effect of ZIP on sensitization when given after induction is that the mechanism of ZIP may be different when given pre-training compared to post-training. It is possible there is a shift to the right in the dose-effect curve for ZIP given post- vs. pre-training. While we used the standard dose of ZIP in the current study, in future studies, it would be interesting to examine the effects of a higher dose of ZIP on sensitization when given after induction; however, it is possible there would be nonspecific effects at higher doses. In the future it would be interesting to compare the effects of a single post-induction ZIP infusion and continuous ZIP infusion on AMPAR density. It is possible, in our experiments, that continuous ZIP infusion reduced post-synaptic AMPAR density below a critical threshold necessary to sustain memory, while the single, post-induction infusion did not (despite using the same total dose of ZIP). Similarly, it is also possible that inhibiting aPKCs prior to training impairs AMPAR insertion or that the newly inserted AMPARs are more vulnerable to the effects of ZIP, potentially because of a difference in sub-unit composition. A more detailed analysis of the type of AMPARs affected by pre- vs. post-training infusions could help to tease apart these explanations. Another alternative is that the neural adaptations produced by a nonassociative, drug-related memory may be more enduring than those in associative memory or the mechanisms may only partially overlap(Robinson and Kolb, 1997; Carmack et al., 2013). A recent study conducted by Carmack and colleagues (2013), using the NMDA receptor antagonist CPP, found that NMDARs were not essential for the induction of sensitization, whereas NMDARs were essential for the formation of place preference. A study conducted by Cai et al. (2011) was one of the few studies to examine the effects of aPKC inhibition on nonassociative memory. In this study, both ZIP and chelerythrine were found to disrupt long-term sensitization of the gill-withdrawal reflex in *Aplysia*, even when given 7 days after training. While both the current study and the Cai et al. study examine the effects of inhibition of aPKCs on sensitization, the mechanisms underlying each of these forms of sensitization is quite different.

AMPAR trafficking is believed to mediate the downstream effects of PKMζ and PKCλ (Ling et al., 2006; Yao et al., 2008; Migues et al., 2010). PKMζ has been reported to enhance AMPA-mediated mEPSCs and application of the synthetic peptide GluR2_3Y_ effectively prevented the endocytosis of GluR2 AMPAR subunits and prevented the deficit in fear memory typically produced by PKMζ inhibition (Ling et al., 2006; Migues et al., 2010). Similarly, PKCλ also affects AMPAR trafficking. Inhibition of PKCλ blocked the LTP-induced enhancement of post-synaptic responses of GluA1 and GluA2 and post-synaptic AMPARs, mEPSCs, and EPSC magnitude are reduced by application of ZIP or PKCλ knockdown (Ren et al., 2013). Our data support and extend previous findings, which suggest the effects of PKMζ and PKCλ are mediated by AMPARs, to cocaine-induced sensitization. In future studies, an interesting comparison would be to examine the effects of ZIP on both sensitized and nonsensitized brain tissue, but for the purposes of this experiment we were primarily concerned with any differences in AMPAR density in cocaine-sensitized animals exposed to ZIP vs. nonZIP.

While ZIP was initially believed to exert its effects on plasticity and memory by selectively inhibiting PKMζ, emerging evidence suggests at concentrations of at least 2 μ M, the peptide acts on PKCλ as well (Ren et al., 2013); this likely accounts for the controversial findings obtained from mice with a deletion of the *Prkcz* gene (Lee et al., 2013; Volk et al., 2013). ZIP still effectively reversed LTP and cocaine-induced place preference in these mice despite the absence of PKMζ (Lee et al., 2013; Volk et al., 2013). Both lines of PKCζ /PKMζ knockout mice exhibit levels of PKCλ that do not differ from controls (Lee et al., 2013; Volk et al., 2013). We found that ZIP effectively impaired nonassociative addiction-related memory and membrane-bound AMPAR expression, but future work will be needed to directly assess the extent to which ZIP exerts its effects on PKMζ, PKCλ or both atypical PKCs. Additional future work will be needed to mitigate the discrepancy between the post-training effects of ZIP on AMPAR density and behavior. As mentioned above, it is possible that a higher dose of ZIP is needed to disrupt AMPAR expression enough to disrupt established sensitization. In the current study we examined global AMPAR density, while future work will examine AMPAR density in a region specific manner.

## General conclusion

In summary, we found that atypical PKC isoforms play a critical role in cocaine-induced locomotor sensitization and addiction. Future work should further explore the differences between traditional forms of associative memory and nonassociative addiction related memory. These differences may elucidate how certain forms of memory may become pathological. Taken together, these findings support a critical role for the atypical PKCs, PKMζ, and PKCλ in cocaine-induced sensitization and therefore in mediating the transition from casual to pathological drug use.

## Author contributions

Kristin K. Howell, Bradley R. Monk, Stephanie A. Carmack, and Oliver D. Mrowczynski performed experiments. Kristin K. Howell, Bradley R. Monk, and Stephan G. Anagnostaras analyzed data. Kristin K. Howell, Bradley R. Monk, and Stephan G. Anagnostaras prepared the figures and wrote the manuscript. Robert E. Clark contributed resources for the measurement of AMPARs. All authors contributed to experimental design and edited the manuscript.

### Conflict of interest statement

The authors declare that the research was conducted in the absence of any commercial or financial relationships that could be construed as a potential conflict of interest.

## Supplementary material

We conducted a place preference test following training for each of the experiments. We did not however explore the effects of ZIP while on-board during place preference. Thus, we cannot make any strong conclusions regarding the effects of ZIP on place preference.

Supplementary Figure S1 **Conditioned place preference assessment. (A)** Place preference was examined for all experiments. In Experiment 1 mice that had previously received cocaine (Veh/Coc, ZIP/Coc) showed place preference whereas mice that had received vehicle (Veh/Veh, ZIP/Veh) did not [*F*_(3, 32)_ = 15.048, *p* < 0.01]. Continuous ZIP did not affect place preference (Veh/Coc, ZIP/Coc, *p* = 0.45). **(B)** Experiment 2. Chelerythrine did not disrupt place preference [*F*_(1, 15)_ = 3.121, *p* = 0.10]. **(C)** An infusion of ZIP given prior to any cocaine administration did not impair place preference [*F*_(1, 15)_ = 1.440, *p* = 0.24]. (**D**) Place preference remained intact when ZIP was given after the induction of sensitization [*F*_(1, 15)_ = 0.023, *p* = 0.88].
