# Supplementary figures and images for: Inhibition of PKC disrupts addiction-related memory
Source: Front Behav Neurosci. 2014 Mar 7;8:70. doi: 10.3389/fnbeh.2014.00070 (PMC3945752; doi:10.3389/fnbeh.2014.00070)

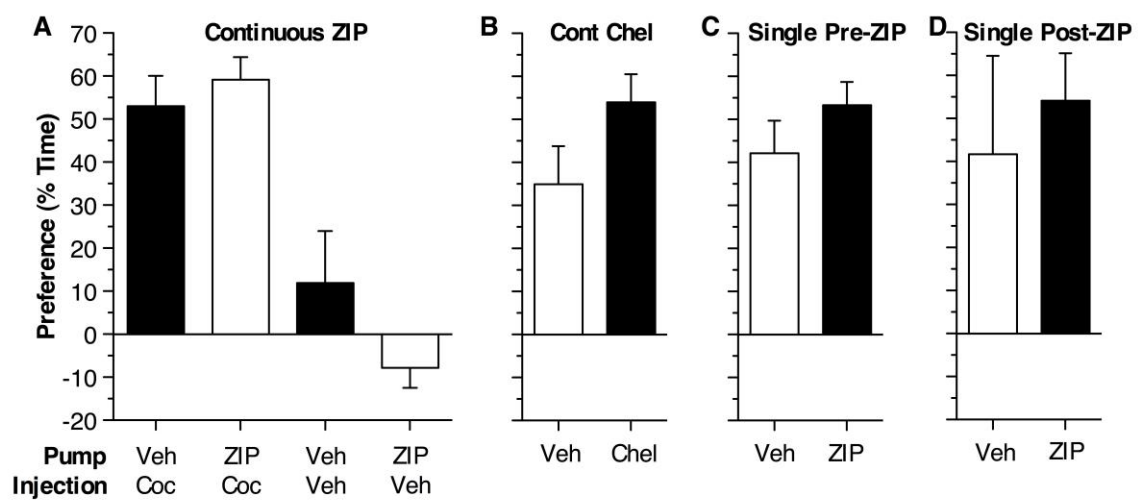

Supplement: Supplementary Figure S1 — Conditioned place preference assessment. (A) Place preference was examined for all experiments. In Experiment 1 mice that had previously received cocaine (Veh/Coc, ZIP/Coc) showed place preference whereas mice that had received vehicle (Veh/Veh, ZIP/Veh) did not [F(3, 32) = 15.048, p < 0.01]. Continuous ZIP did not affect place preference (Veh/Coc, ZIP/Coc, p = 0.45). (B) Experiment 2. Chelerythrine did not disrupt place preference [F(1, 15) = 3.121, p = 0.10]. (C) An infusion of ZIP given prior to any cocaine administration did not impair place preference [F(1, 15) = 1.440, p = 0.24]. (D) Place preference remained intact when ZIP was given after the induction of sensitization [F(1, 15) = 0.023, p = 0.88]. [file Presentation1.PDF]
